# A deep fusion‐based vision transformer for breast cancer classification

**DOI:** 10.1049/htl2.12093

**Published:** 2024-10-23

**Authors:** Ahsan Fiaz, Basit Raza, Muhammad Faheem, Aadil Raza

**Affiliations:** ^1^ Department of Computer Science COMSATS University Islamabad (CUI) Islamabad Pakistan; ^2^ School of Technology and Innovations University of Vaasa Vaasa Finland; ^3^ Department of Physics COMSATS University Islamabad (CUI) Islamabad Pakistan

**Keywords:** artificial intelligence, breast cancer, classification, deep learning, histopathology images, machine learning

## Abstract

Breast cancer is one of the most common causes of death in women in the modern world. Cancerous tissue detection in histopathological images relies on complex features related to tissue structure and staining properties. Convolutional neural network (CNN) models like ResNet50, Inception‐V1, and VGG‐16, while useful in many applications, cannot capture the patterns of cell layers and staining properties. Most previous approaches, such as stain normalization and instance‐based vision transformers, either miss important features or do not process the whole image effectively. Therefore, a deep fusion‐based vision Transformer model (DFViT) that combines CNNs and transformers for better feature extraction is proposed. DFViT captures local and global patterns more effectively by fusing RGB and stain‐normalized images. Trained and tested on several datasets, such as BreakHis, breast cancer histology (BACH), and UCSC cancer genomics (UC), the results demonstrate outstanding accuracy, F1 score, precision, and recall, setting a new milestone in histopathological image analysis for diagnosing breast cancer.

## Graphical abstract

The proposed model employs fusion at two different levels. Firstly, image fusion is performed during the data preprocessing stage. The convolutional neural network base VGG16 model is utilized for feature extraction, and these features are then fused into a vision transformer before being passed through a multi‐layer perceptron.

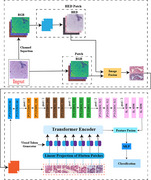

## 1 INTRODUCTION

Cancer, particularly breast cancer, poses a significant global health threat, with 19.3 million new cases reported in 2020 and 680,000 women succumbing to the disease [1]. Early detection is challenging due to subtle symptoms and the small size of lumps, emphasizing the need for a robust screening system [2]. Breast cancer can be either malignant or benign, with malignant cases being life‐threatening and benign ones non‐cancerous. Doctors typically rely on X‐rays and microscope images for analysis, but manual interpretation is highly challenging [3]. Various diagnostic methods, such as mammography and staining techniques like haematoxylin‐eosin, aid in analysing tissue conditions [4].

Computer‐aided diagnostic (CAD) systems play a crucial role in disease diagnosis by reducing workload and minimizing errors [5]. Despite advancements in CAD systems, diagnosing breast cancer remains difficult due to the disease's high prevalence, time‐consuming procedures, and the variability of expert opinions [6]. Deep learning, particularly convolutional neural networks (CNNs), has emerged as a powerful tool for histological image classification, helping to speed up diagnosis, enhance the effectiveness of screening, and improve diagnostic consistency among pathologists [7, 8, 9, 10].

CNNs use convolution kernels to extract features, but class imbalance remains a challenge. Generative adversarial networks (GANs) have been applied to augment data samples, though GAN‐generated data may not always accurately represent real‐world scenarios [11, 12]. To address these issues, researchers have introduced attention‐based high‐order deep networks, which combine attention mechanisms with high‐order statistical representations to capture more discriminating features in breast cancer images [13].

The Transformer architecture, based on attention mechanisms, has also proven effective in extracting global features from images, making it useful for image classification [14]. CNN and Vision Transformer models, originally designed for natural image classification, have shown reasonable performance on histopathology images. Still, CNN‐based models struggle to detect the relative position patterns between cells and layers composed of clustering cells. Additionally, cell‐layer level patterns are relatively small and randomly distributed in different locations, limiting model accuracy, precision, recall, and F1 score.

The proposed research introduces a fusion‐based model that explores the combination of original and normalized images. It also investigates the impact of fused features extracted from CNN and Vision Transformer models to enhance diagnostic outcomes. The main contributions of this paper are outlined below:

1. We have worked extensively on how the fusing of the stain‐normalized and RGB histopathological images will be useful. The rationale behind this approach to image fusion is to seek out complementary information from these two image types and to improve model performance in order to learn complex features related to cancerous tissues.
2. A state‐of‐the‐art deep learning hybrid model was proposed. The deep‐fused Vision Transformer, also known as DFViT, was designed for cancerous cell classification. DFViT unifies CNN and transformer‐based architectures into a much stronger and more versatile model for handling both local and global features in medical images.
3. Extensive testing on one binary and two multiclass benchmark datasets has been done using the DFViT model. The model outperforms existing models by getting better accuracy, precision, recall, and F1‐score, hence proving to be effective for breast cancer classification.
4. We conducted an in‐depth investigation into the impact of merging feature sets from the VGG16 CNN and Vision Transformer. This strategy explores how combining the low‐level spatial features of CNNs with the high‐level contextual features of transformers enhances classification performance.

The primary objectives of this research are to utilize the Vision Transformer (ViT) and CNN‐based model in creating a novel deep fusion‐based model for classifying breast cancer, to evaluate the performance of the proposed model on three distinct datasets, each representing a different facet of breast cancer classification, and to address challenges and conduct comparisons with existing models.

The organization of this paper is as follows: In Section 2, we provide an overview of the deep learning models employed in classification tasks. Moving on to Section 3, the distinctive aspects of the proposed DFViT model are detailed. Section 4 explains the three datasets utilized in this study. Section 5 is dedicated to presenting and examining the outcomes of our experimental models. Section 6 discusses the limitations of the proposed model. Finally, Section 7 offers a summary of the study, while Section 8 outlines potential directions for future research.

## 2 RELATED WORK

Researchers in the field of histopathology image analysis utilized various deep‐learning models to enhance the classification of cancerous cells. Ruifrok et al. [15] pioneered the use of colour deconvolution to separate stains in tissue cell images. Neural network models, including AlexNet [16], VGG [17], ResNet [18], and EfficientNet [19], became popular for histopathology image classification. Transfer learning was employed, with ResNet50 and Inception‐V3 achieving high accuracy in breast cancer classification [20]. However, challenges arose in classifying histopathological images due to staining characteristics [21]. Graham et al. [22] introduced LeViT, a deep learning model combining CNN architecture with a vision transformer, but it showed reduced performance on different datasets. GasHis‐Transformer [23] and Vision Transformer (ViT) [24] were proposed for gastric cancer and achieved state‐of‐the‐art performance. Modified AlexNet [25] exhibited good accuracy in locating patches but had limitations in generalization. Bayramoglu et al. [26] proposed single and multi‐task CNNs for cancer detection, achieving accuracy rates of 83.39% and 83.63%, respectively.

Ratiher et al. [27] developed a hybrid model with an autoencoder‐decoder for cancerous cell classification. Bardou et al. [28] compared deep learning traits with hand‐crafted ones, noting performance deterioration for simpler images. Sharma et al. [29] presented CNN architecture for automated breast cancer histopathology image classification. Alkassar et al. [30] used DenseNet and Xception for colour separation enhancement, achieving optimal performance with a multi‐classifier method. Thapa et al. [31] proposed a CNN‐based system with patch‐wise classification and majority voting for refinement. Shallu et al. [32] demonstrated VGG‐16 with logistic regression's superiority on a binary class dataset but struggled with multi‐class datasets.

However, classifying breast cancer in histopathology images using CNNs posed several challenges. CNNs were limited in capturing stain variations that indicate specific tissue components, often leading to poor interpretation of stain‐specific features. They could not also study the spatial relationships between cells and tissue layers, which are critical for detecting cancer patterns. Additionally, CNNs were prone to overfitting due to the small size of histopathological datasets, reducing their generalization capability. Moreover, CNNs depend solely on local features and often fail to capture the global structure of an image, which is essential for tissue analysis. High‐resolution images, typically obtained in histopathology, also presented a computational challenge.

Chattopadhyay et al. [33] proposed a channel attention‐based model for binary classification, while He et al. [34] introduced a deconvolution and transformer‐based architecture for breast cancer classification, addressing overfitting issues. Gao et al. [35] developed an instance‐based Vision Transformer for histopathological images, which showed effective results but lower accuracy compared to other models. Transformer architectures were predicted to play a more prominent role in tissue cell image analysis. Krishna et al. [36] introduced the attention branch network (ABN) for interpretable decision support in binary classification. Maleki et al. [37] focused on improving the speed and precision of histopathological image classification using transfer learning and extreme gradient boosting (XGBoost).

Abtan et al. [38] proposed ResNet18 with meta‐heuristic algorithms for breast cancer pathology images, achieving high F‐score but imperfect results in other measures. Kumar et al. [39] developed the SELF framework based on stacked ensemble learning for early‐stage breast cancer classification. Sahu et al. [40] proposed a computer‐aided ensemble method combining a pre‐trained ResNet18 model and SVM for breast cancer diagnosis, incorporating haze reduction techniques and tumour segmentation.

Doe et al. [41] present computing techniques for breast cancer detection, primarily through the use of mammogram images. While it outlines important advancements in CAD systems, the focus is mainly on conventional imaging modalities and mammograms. Jonson et al. [42] outlined computing techniques for breast cancer detection, focusing on mammogram images and CAD systems. Ali et al. [43] applied transfer learning for breast cancer image classification, which aligned with the use of pre‐trained models (e.g. VGG16) in conjunction with Vision Transformers. While their study focused on mammograms, our work advances classification by applying deep fusion to histopathological images, which involve more complex tissue structures. Zang et al. [44] discussed AI approaches in the context of omics data, which shared similarities with image‐based analysis. While omics data processing faced challenges in handling complex features and high‐dimensional data, our work addressed similar issues in histopathological images by using deep learning architectures like Vision Transformers and CNNs.

Despite promising results, several studies acknowledge the need for further improvements in classification accuracy and generalization to different datasets. However, results need to be improved.

The summary of the literature review is shown in Table 1.

**TABLE 1:** Summary of literature review.

Author	Description	Dataset	Performance metrics
Albashish et al. [21]	CNN‐based VGG16 model using pre‐trained weights	BreakHis	Accuracy Sensitivity
Chen et al. [24]	Vision Transformer is a backbone. It extracts features. Resnet is used for classification	HE‐GHI‐DS	Precision, Recall F1‐score, Accuracy
Spanhol et al. [25]	CNN base model is used to extract features. The SVM is used to classify the images.	BreakHis	Accuracy
Bayramoglu et al. [26]	Merge images of different magnifications. CNN base model performs classification	BreakHis	Accuracy
Pratiher et al. [27]	The model is based on auto encoder‐decoder using manifold learning for the classification	BreakHis	Accuracy Sensitivity Specificity
Bardou et al. [28]	CNN is used for feature extraction and classification	BreakHis	Accuracy Precision Recall F1‐score
Hu et al. [34]	Vision Transformer base classification model	BreakHis, Bach UC	Accuracy, Precision Recall, F1‐score
Gao et al. [35]	Instance‐based Vision Transformer	Papillary Renal Cell Carcinoma subtyping	Accuracy
Krishna et al. [36]	Attention branch network that combines with customized DarkNet19	BreakHis	Accuracy, Recall, Precision, F1‐score
Maleki et al. [37]	XGBoost with Transfer Learning	BreakHis	Accuracy, Recall, Precision, F1‐score
Abtan et al. [38]	Utilized ResNet18 with heuristic Algorithms	BreakHis	Accuracy, Recall, Precision, F1‐score
Kumar rt al. [39]	Stacking ensembles techniques and other classifiers like the random forest, Ada Boost, Gradient boosting and KNN9	BreakHis WBCD	Accuracy, Recall, Precision, F1‐score
Sahi et al. [40]	ResNet18 for feature extraction and SVM for classification	BreakHis	Accuracy, Precision Recall, F1‐score

We delve into the impact of fusing stain and RGB histopathological images on transformer architectures and CNN models in this paper. Furthermore, we present novel models designed to comprehensively leverage the fusion technique in two distinct stages. To our knowledge, this study represents the inaugural attempt at fully integrating fused histopathology images to extract features from both CNNs and multi‐head attention elements. Subsequently, the resulting fused vector is employed for classification purposes.

## 3 METHODOLOGY

**FIGURE 1:**
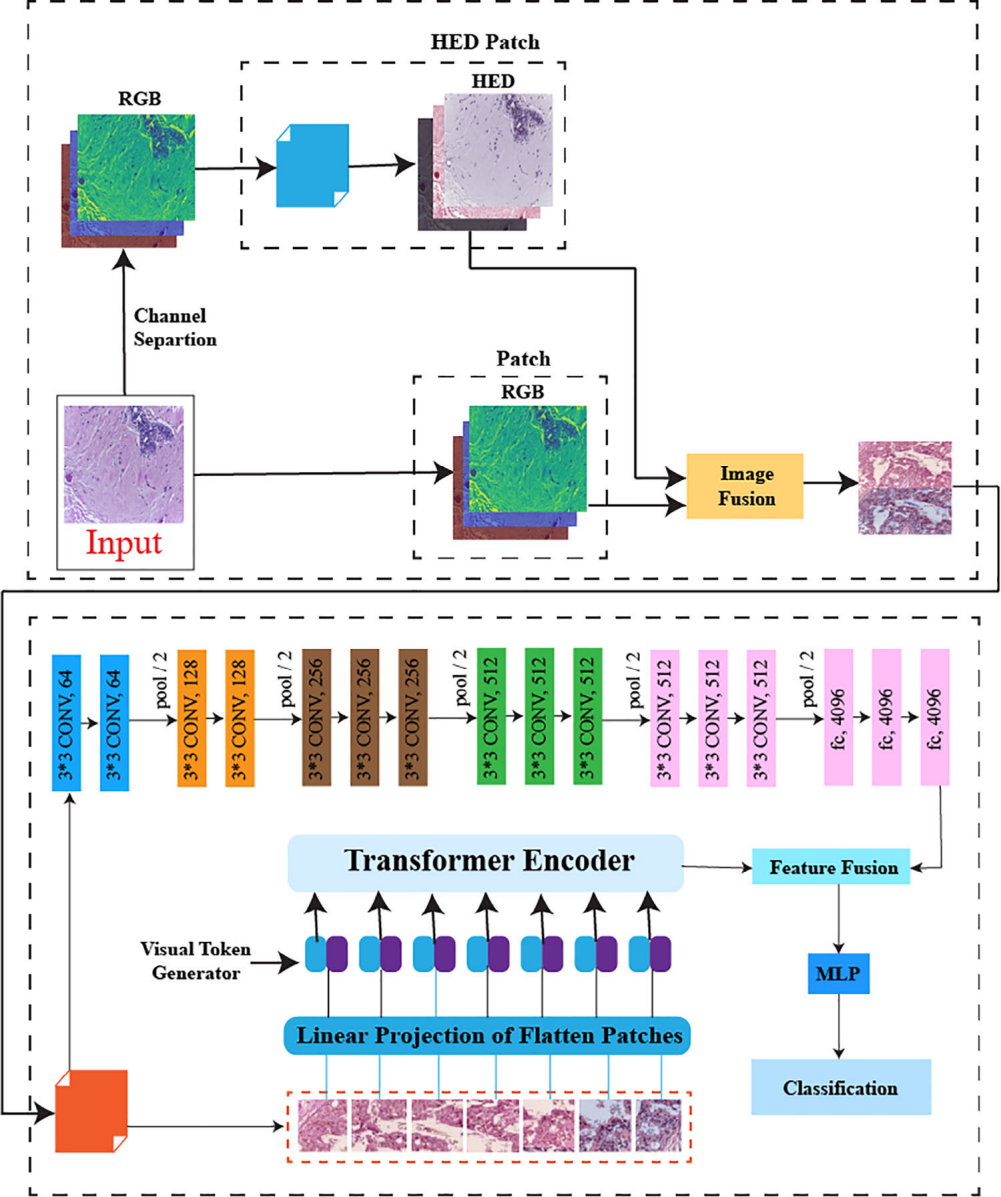
Proposed mode.

In this study, we propose a novel classification methodology for breast cancer histopathological images by integrating CNN and transformer architectures. The following section outlines the key steps of the proposed approach. The schematic of the proposed classification methodology is depicted in Figure 1. This method comprises three primary phases: fusion of stain‐normalized and RGB histopathological images, feature extraction through CNN‐based and transformer models, fusion of the resulting feature vectors, and subsequent classification. In the initial step, stain normalization is applied to the original image, followed by concatenation of the normalized image with the original one. The concatenated image is then processed by both VGG16 and the Vision Transformer. Feature vectors are extracted and passed into the Vision Transformer's multi‐layer perceptron (MLP) head, which ultimately performs classification using an MLP.

In the proposed model, deep fusion occurs before the classification stage by combining features from CNNs, which excel at capturing local patterns, and Vision Transformers, which capture global context. This fused feature set is then processed through a multi‐layer perceptron (MLP) for final classification. Such deep fusion enhances the analysis of the complex and heterogeneous structures present in breast cancer histopathology images, leading to improved accuracy, precision, recall, and overall classification performance. The following sections elaborate on the individual steps of this proposed methodology.

The algorithm for the model is shown below.

**Table:** 

Algorithm: Stain‐Normalized and RGB Image Fusion for Breast Cancer Classification
Input:
‐ Stain‐normalized histopathological image (stain_normalized_image)
‐ RGB histopathological image (rgb_image)
Output:
‐ Predicted class label (predicted_class)
1. Fusion of Images:
1.1 Combine stain‐normalized and RGB images:
‐ Fused_image = CombineImages(stain_normalized_image, rgb_image)
2. Feature extraction:
2.1 CNN‐based feature extraction:
‐ Extract CNN_features from Fused_image using a trained CNN based VGG16 model.
2.2 Transformer‐based feature extraction:
‐ Extract Transformer_features from Fused_image using a trained transformer model.
3. Fusion of feature vectors and classification:
3.1 Feature fusion:
‐ Combine CNN_features and Transformer_features, e.g. by concatenation.
3.2 Classification:
‐ Use a multi‐layer perceptron (MLP) classifier to predict the class label:
‐ predicted_class = MLP_Classifier(Fused_Features)
4. Output:
‐ Return the predicted_class as the final classification result.
End of algorithm

### 3.1 Image fusion

**FIGURE 2:**
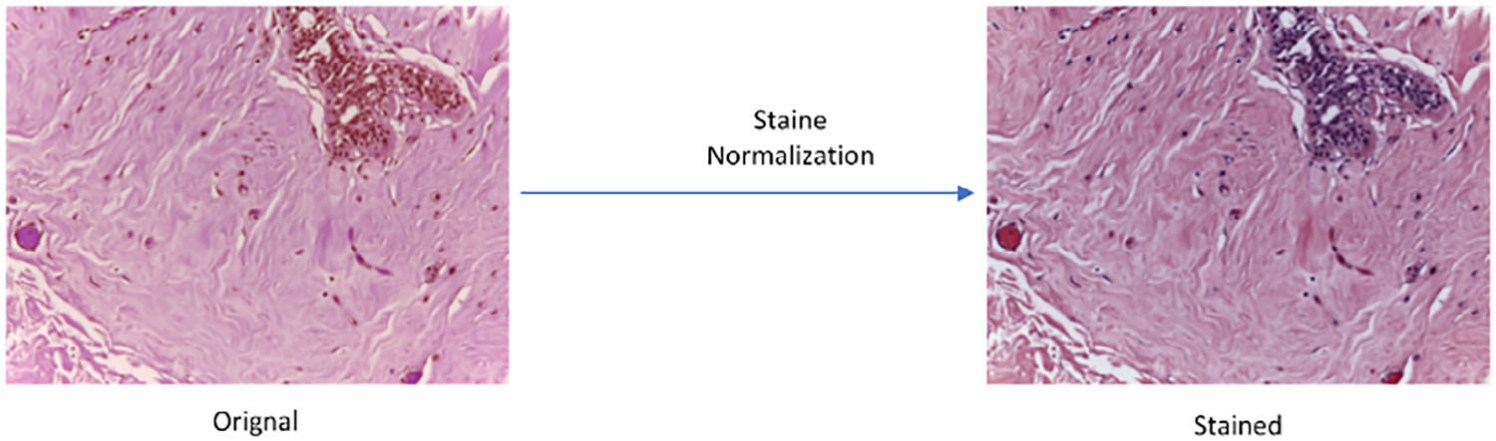
Effect of stain normalization.

Image fusion is a technique that involves concatenating two or more different colour scheme images to create a composite image incorporating data from the original images. Histopathology images contain stain properties, so we first need to perform a stain normalization process. Stain normalization changes image colours and brightness to a common standard. Stain normalization fixes this by changing histopathological images to a common colour style, making stains look the same in all images. It works by adjusting each image's colours to match a standard reference. This helps machine learning models concentrate on disease‐related traits instead of getting confused by staining differences. In short, stain normalization is crucial for reliable machine learning in medical diagnosis and research, preventing colour differences from affecting the accurate analysis of diseases. The effect of stain normalization is shown in Figure 2.

**FIGURE 3:**
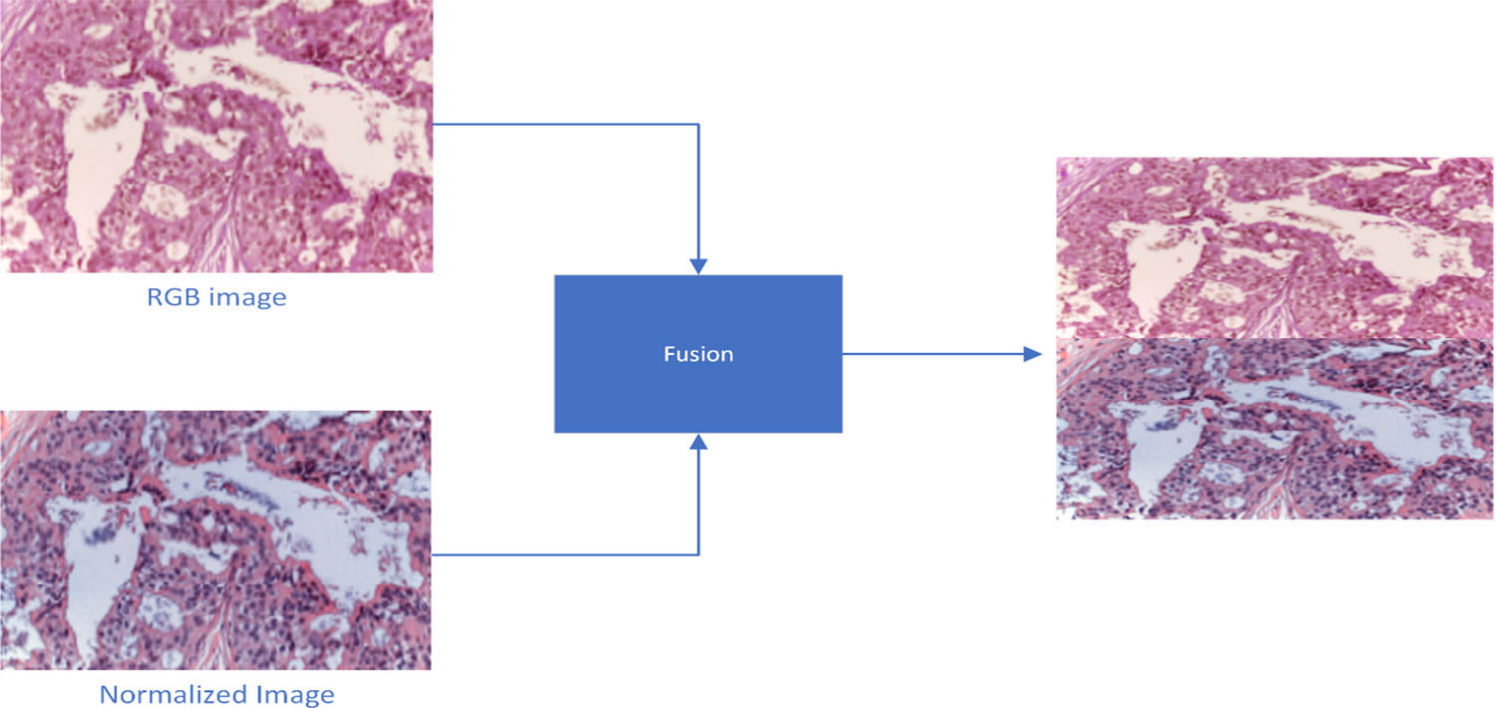
Image fusion.

However, this change might cause us to lose important original details, like slight staining differences that could help with diagnosis. Going too far with normalization might erase helpful features we need for careful examination. To mitigate this loss, we fuse the original image with the resultant image generated by the normalization technique, minimizing the loss of features. The process is shown in Figure 3.

Let Ha be the height of the RGB image and Hb is the height of the stained image. The height of the resultant image Hc will be the sum of Ha and Hb.

(1) Hc=Ha+Hc

The width of image C remains the same as the width of image A:

(2) Wc=Wa

### 3.2 Feature fusion

Feature fusion is the integration of features from distinct layers or branches. It is a common component in current network architecture. It is often implemented using simple techniques like summation concatenation, and weighted sum. We have two feature vectors X and Y. Let us assume X has *n* features and Y has *m* features. To concatenate them horizontally, we create a new feature vector Z like this:

(3) $$Z\;=\left[X,Y\right]\;$$

The resulting vector Z will have *n* + *m* features.

The study utilizes the CNN‐based VGG16 model for feature extraction, known for its effectiveness in computer vision tasks. VGG16 employs a deep architecture with small (3 × 3) convolution filters, showcasing significant advancements. Predominantly used for classification, it achieves 92.7% accuracy on one thousand classes in the ImageNet dataset and comes pre‐trained with readily available weights. The final classification layer is removed by modifying the model architecture. This involves cutting off the last fully connected (dense) layer and retaining the layers up to the penultimate layer, which outputs feature vectors. This setup enables VGG16 to generate feature embeddings without performing classification. Features are extracted from VGG16 and stored in a vector. The Vision Transformer (ViT) is introduced as an expansion of the transformer architecture. ViT divides input images into patches, termed visual tokens, which are transformed into fixed‐dimension encoded vectors. Each ViT encoder comprises Layer Normalization to adapt to image variations, and a multi‐head attention network (MSP) for critical attention mechanisms, allowing the model to focus on essential areas of the image for learning hierarchies and alignments in the input data. The feature fusion combines the pre‐trained CNN, VGG16, with the Vision Transformer. Moreover, CNNs are really powerful for local features and pattern extraction; large pre‐trained models like VGG16 have been trained on huge datasets‐for example, ImageNet‐and hence, the model can leverage these learned features. Therefore, it helps avoid training the Vision Transformer from scratch, which normally requires large datasets. It leverages the strengths of CNNs in localized and low‐level feature extraction and the global feature extraction provided by Vision Transformers by fusing the features from both CNNs (VGG16) and Vision Transformers. This decreases the dependency of the Vision Transformer on large datasets by providing it with a rich set of pre‐learned features that make it very effective even for smaller datasets.

When performing feature fusion, the process generates a large vector set that combines features from different sources, which can lead to an increase in dimensionality and potential overfitting. To address this issue, we use a multi‐layer perceptron (MLP) with dropout regularization. The dropout technique randomly omits a subset of neurons during training, effectively reducing the network's complexity and mitigating overfitting. This approach helps manage the large feature vectors produced by fusion, ensuring that the model remains robust and generalizes well to new data.

### 3.3 Multi‐layer perceptron for classification

The MLP is a neural network designed for classification tasks, featuring fully connected layers where every neuron in one layer is linked to every neuron in the next layer. The network utilizes the Gaussian error linear unit (GELU) activation function to introduce non‐linearity, crucial for capturing complex patterns in data. For multi‐class classification, the final output is transformed using the SoftMax function, converting numerical outputs into probabilities that represent the likelihood of each class. In binary classification, the Sigmoid function is employed independently for each output neuron, suitable for binary decisions. The MLP processes a fused vector, a combination of various information pieces, using GELU, and applies SoftMax or Sigmoid based on the classification scenario to determine the most likely category, facilitating informed decision‐making based on probabilities.

## 4 DATASETS

**FIGURE 4:**
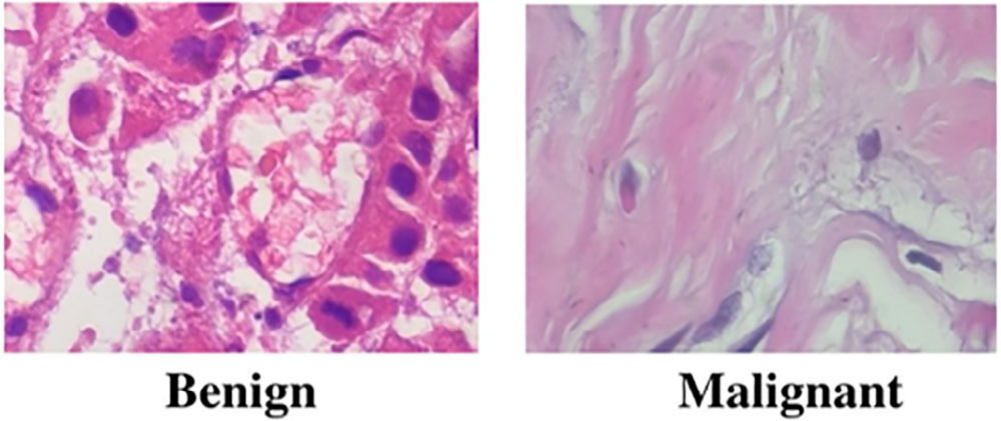
Sample of BreakHis dataset.

We are utilizing three different datasets to validate our model. The BreakHis [45] dataset is a frequently utilized resource for studying image classification in breast cancer histopathology. This dataset comprises 7909 histopathology images collected from eighty‐two distinct patients. The images are captured at four different magnifications (40, 100, 200, and 400), with an average size of 460 × 700 pixels per image. The dataset contains two major categories: malignant and benign. Specifically, it has 2480 benign tumour images from twenty‐four patients and 5429 malignant tumour images from 58 people. Figure 4 shows the sample of BreakHis dataset.

**FIGURE 5:**
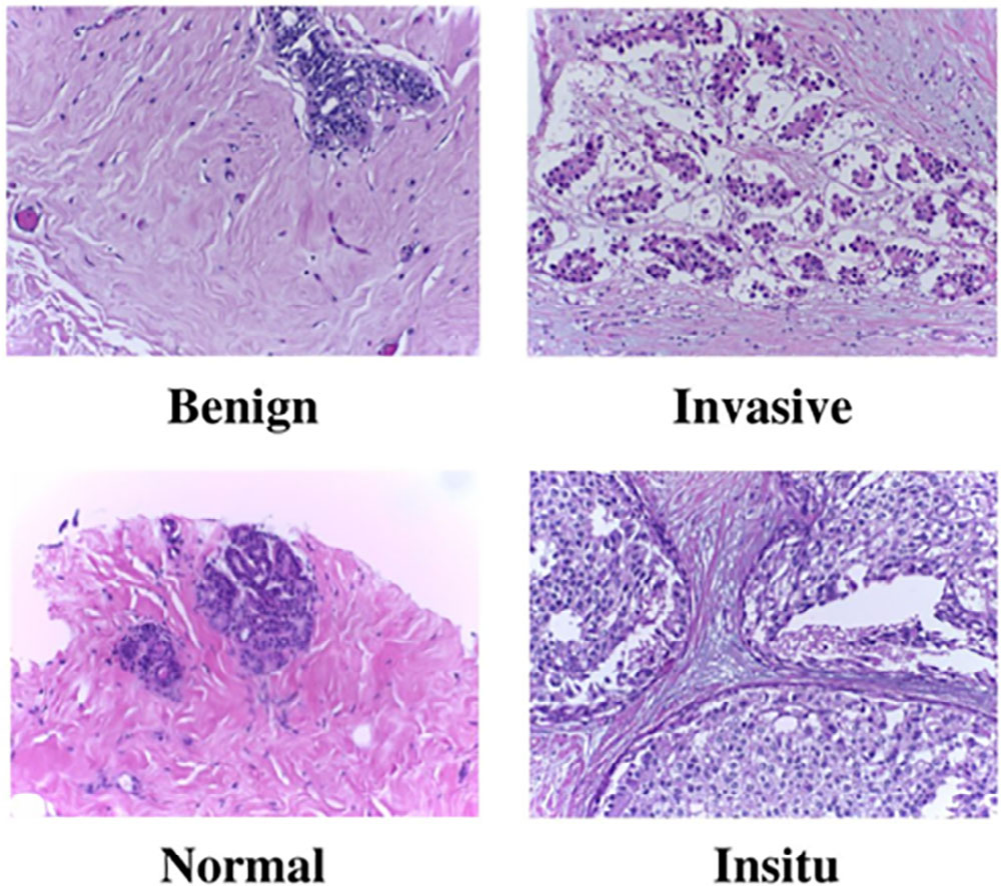
Samples of the BACH dataset.

The second dataset used for validation is the BACH dataset [46], which is divided into two parts. The first part consists of 400 breast cancer histopathology microscope images, categorized into four groups: normal, carcinoma in situ, benign, and invasive carcinoma with a hundred images in each class. The other section involves pixelated labels of whole‐slide breast histology images, but we utilize only the first part. Figure 5 shows the sample of the BACH dataset.

**FIGURE 6:**
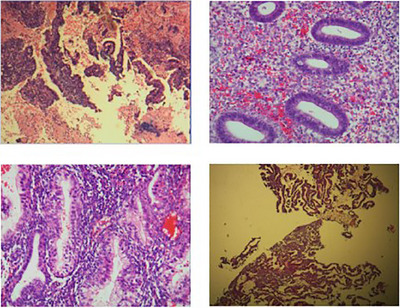
Sample of normal endometrium.

**FIGURE 7:**
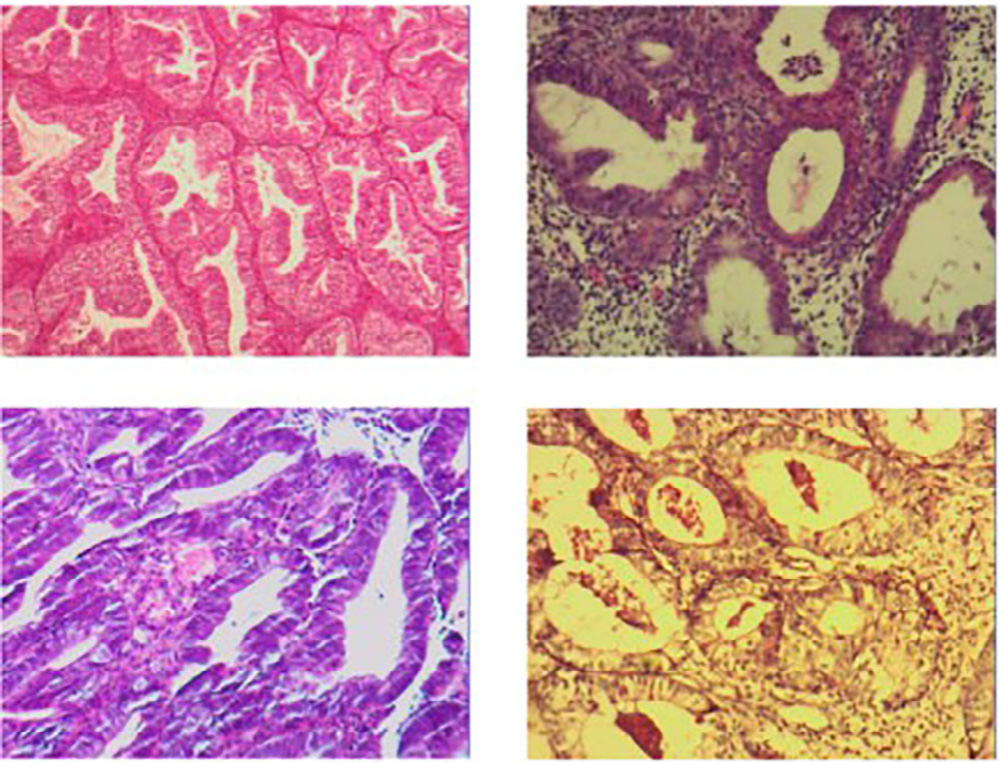
Sample of endometrial hyperplasia.

**FIGURE 8:**
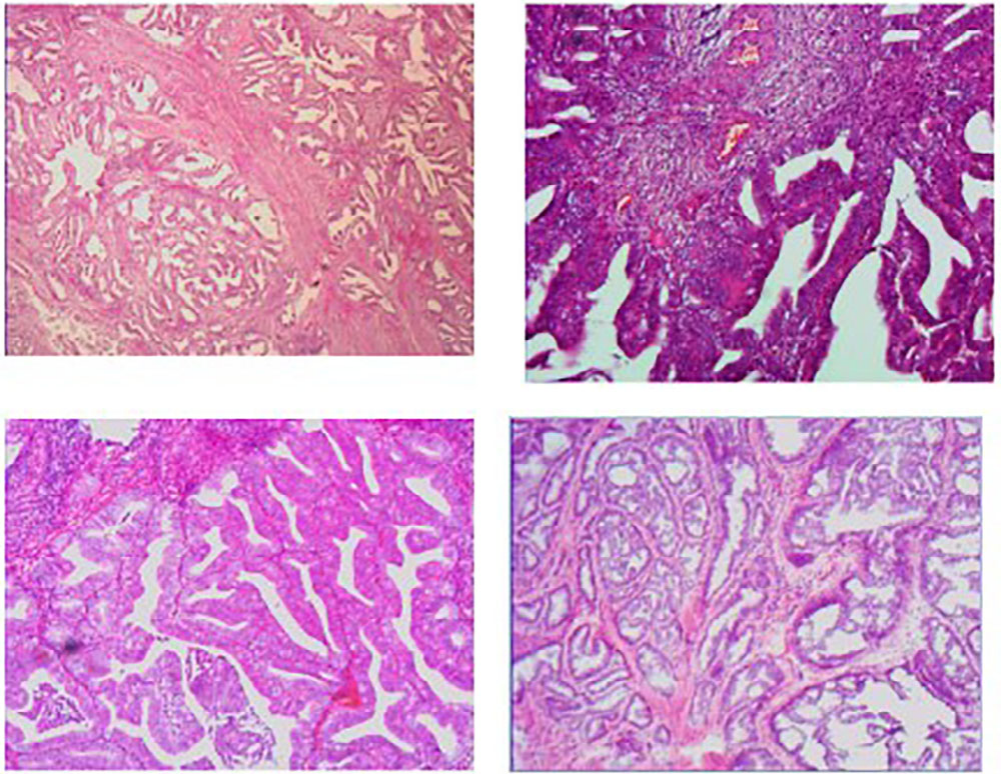
Sample of endometrioid cancer.

**FIGURE 9:**
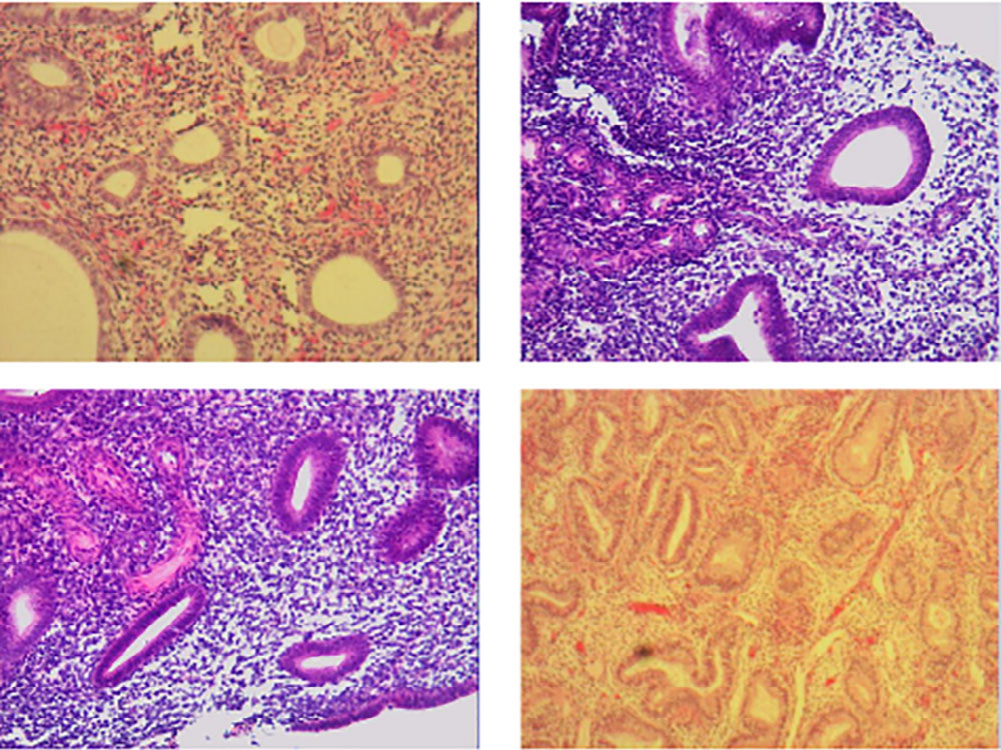
Sample of endometrial polyps.

The third dataset we utilized is the UC dataset [47], which comprises 500 endometrial specimens, each labelled with one of the four categories: normal endometrium, endometrial hyperplasia, endometrioid cancer, and endometrial polyps. Figures 6, 7, 8, 9 show the sample, respectively. These specimens form part of the endometrial dataset in the UC dataset. Figure 6 shows the sample of the UC dataset.

Datasets used in BACH and UC for multi‐class classification problems involve several categories for breast cancer or endometrial conditions, such as benign, in situ, invasive, and normal. However, the model has to differentiate between more than two classes. In binary classification, data related to BreakHis includes two classes, generally malignant versus benign, which simplifies the problem into distinguishing between cancerous and non‐cancerous samples. The variation in multi‐class datasets increases the level of challenge compared to binary classification. The summary of all three datasets is described in Table 2.

**TABLE 2:** Dataset information.

Dataset	Classes	Classes	Number	Size	Total	Tissue parts
BreakHis	2	Malignant	5429	700 × 460	7909	Breast
		Benign	2480			
Bach	4	Benign	100	2048 × 1536	400	Breast
		InSitu	100			
		Invasive	100			
		Normal	100			
UC	4	EA	535	640 × 480	3302	Endometrium
		EH	798			
		EP	636			
		NA	1333			

## 5 EXPERIMENT SETUP

This section discusses the datasets, experimental settings, and baseline models that were used to train and assess the suggested model. For our implementation, we utilized TensorFlow and conducted our experiments on a Colab Notebook with a 12GB NVIDIA Tesla K80 GPU. The model was compiled using the Adam optimizer with sparse categorical cross‐entropy as the loss function, suitable for multi‐class classification. For feature extraction, we use a pre‐trained VGG16 with an input image size of 225 × 225, retaining its default parameters. The Vision Transformer (ViT) also operates with the same image size of 225 × 225, with a learning rate of 0.0001 and 50 epochs. For classification, the multi‐layer perceptron (MLP) consists of three hidden layers with 512 units each, uses ReLU as the activation function, and incorporates a dropout rate of 0.3. Accuracy was used as the evaluation metric during training and validation. The proposed model was tested based on several key metrics, including accuracy, recall, precision, and F1‐score. These metrics will evaluate different dimensions of the performance in classification: accuracy describes the general correctness of the classification, precision refers to the ratio between true positives and all the predicted positive cases, recall reflects the capability of the model to detect all relevant instances, whereas the F1‐score is a balanced version of both. The model outperforms the different existing CNN‐based models in the analysis on breast cancer classification. It achieved a higher accuracy and F1‐score, which is indicative of its overall good classification performance with a more balanced detection of both the positive and negative instances of the dataset.

### 5.1 Result and comparison

In this section, we thoroughly evaluate the performance of the proposed model by subjecting it to a series of challenging tests. The suggested model's performance is compared to different baseline models, enabling a comprehensive study and evaluation of its effectiveness. Our analysis involves using the BreakHis dataset, the BACH dataset, and the UC dataset, where we assess the proposed model's performance against existing state‐of‐the‐art classification methods. Among the datasets, one is binary, while the other two are multiclass. We conduct quantitative comparisons between the predictions made by the suggested DFViT on unseen test images and those made by other state‐of‐the‐art models, including DeconVit, Resnet, VGG16, and VGG19. In contrast to traditional classification techniques, our proposed model, known as Deep Fusion‐based ViT, demonstrates the ability to accurately identify the provided images in the subsequent sections. This will be discussed in detail in the following paragraphs.

#### 5.1.1 Result on BACH

**FIGURE 10:**
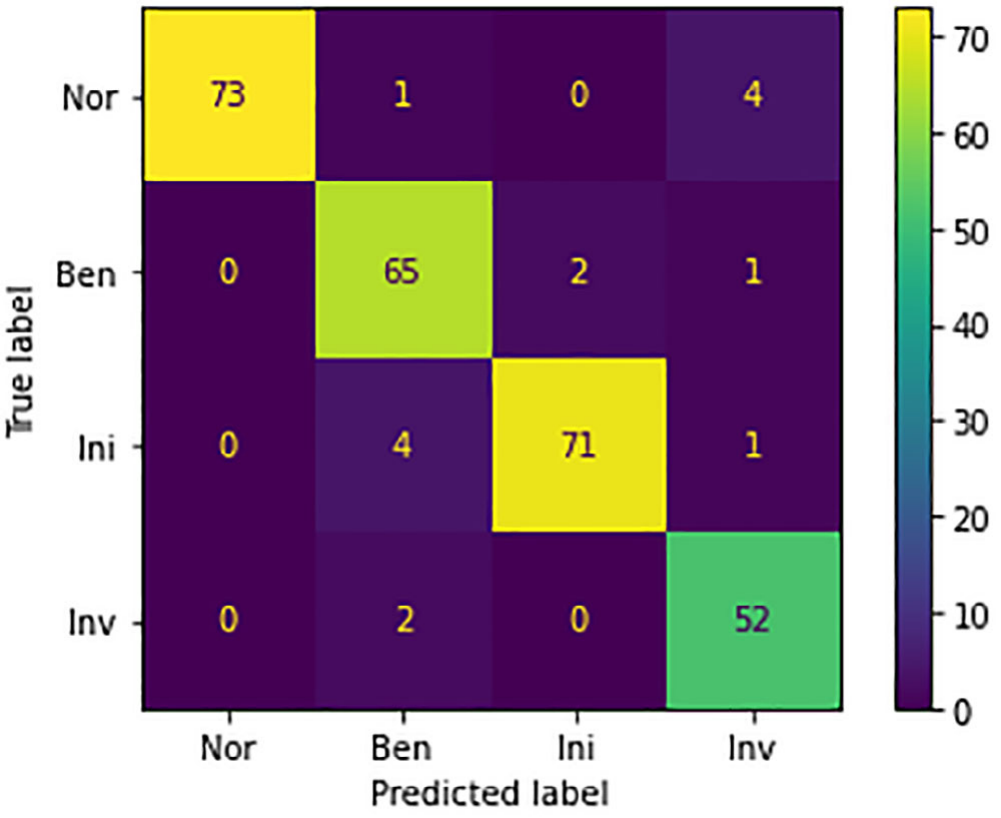
Confusion metrics on BACH.

The study evaluates the model's performance using the BACH dataset, which contains labelled images for assessing its abilities. The dataset includes four classes: benign (1), normal (0), in situ (2), and invasive (3). Key metrics like precision, accuracy, F1‐score, and recall are analysed for each class, providing a detailed view of the model's performance, the normal class exhibits room for improvement, with five instances of mispredictions out of 78. Conversely, the benign class showcases high accuracy, accurately predicting 65 out of 68 instances. The model excels in the invasive class, correctly predicting 52 out of 54 instances, demonstrating proficiency. In the in situ class, the model accurately predicts 71 out of 76 instances, establishing reliability across diverse class scenarios. Figure 10 presents the confusion metrics.

The model's performance is impressively robust, achieving high accuracy levels across all classes. In our analysis, we found interesting results. For the normal class, which had 78 instances, there were five cases where our predictions were off, suggesting that the model could improve in recognizing this class. On the other hand, in the benign class with 68 instances, our model accurately predicted 65 of them, showcasing its effectiveness. The model's performance was especially strong in the invasive class. It correctly predicted 52 out of 54 instances, highlighting its proficiency in this area. Similarly, in the in situ class, the model's ability was evident as it accurately predicted 71 out of 76 instances, demonstrating its reliability across different class scenarios. This success is visually represented through an image. The confusion metrics on the BACH dataset are shown in Figure 10. Furthermore, to provide a comprehensive picture of our model's performance, we have included a detailed comparison with other models on the BACH dataset in Table 3. This comprehensive validation approach underscores the model's effectiveness and solidifies its reputation as a high‐performing solution for navigating the complexities of the multi‐class BACH dataset.

**TABLE 3:** Description of average results.

Model	Accuracy	Precision	Recall	F1‐score
Resnet	70	83	88	85.47
DenseNet	61	69	70	69.21
VGG16	77	81	83	82.00
VGG19	77	80	79	79.62
BiT‐M	50	61	60	60.66
DeconVit [34]	79	77	75	75.99
Proposed model DFVit	94	98	95	96.48

#### 5.1.2 Results on UC

**FIGURE 11:**
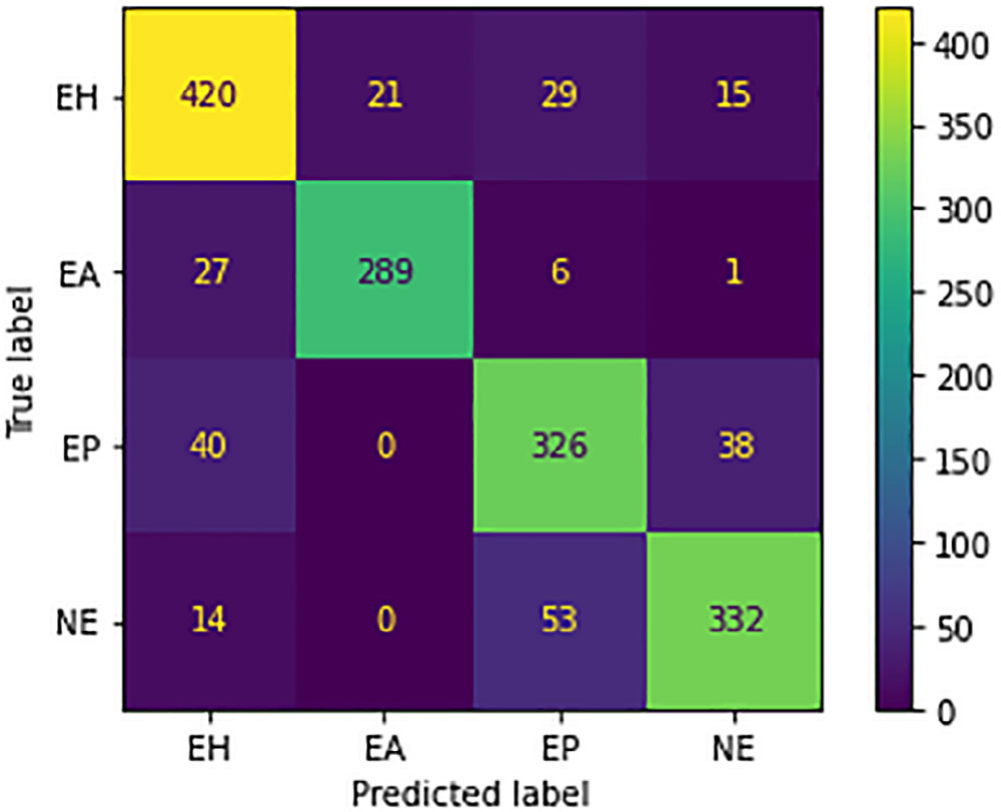
Confusion Metrics on BACH.

The study extensively evaluated the performance of the model using the UC dataset, which contains meticulously labelled images representing four types: endometrial hyperplasia (class 0), endometrioid cancer (class 1), endometrial polyps (class 2), and normal endometrium (class 3). Precision, accuracy, F1‐score, and recall were thoroughly examined for each group, providing a comprehensive understanding of the model's efficacy. Noteworthy results emerged, with the model excelling in predicting endometrial hyperplasia (EH) images (420 accurately predicted), endometrioid cancer (EA) images (289 accurately predicted), endometrial polyps (EP) images (326 accurately predicted), and normal endometrium (NE) images (332 accurately predicted). A comparative analysis with other models, presented in Table 4, further underscored the model's standing. Visual representations of the model's performance were also provided in Figure 11, enhancing the overall confidence in its capabilities, and affirming its reliability in navigating the complexities of the UC dataset across diverse classes.

**TABLE 4:** Description of average results for UC.

Model	Accuracy	Precision	Recall	F1‐score
Resnet	70.1	71	70	70.57
DenseNet	61.5	55	52	53.46
VGG16	77.06	68	58	62.60
VGG19	77.50	58	53	55.35
BiT‐M	50.10	53	49	50.92
DeconVit [34]	81.36	78	76	77.05
Proposed model DFVit	84.56	82	81	81.51

#### 5.1.3 Result on BREAKHis

We validate the performance and gain insights into our model's functioning by employing the BreakHis dataset. This dataset is equipped with labelled images and takes the form of a binary classification challenge, consisting of two distinct classes: 0 for benign and 1 for malignant. Notably, each image arrives in varying resolutions, prompting us to categorize them based on resolution and subsequently tailor our model's training approach. To gauge performance, we meticulously compute precision, accuracy, F1‐score, and recall for each class individually. Additionally, we conduct a comprehensive comparative assessment between our model and other models on the BreakHis dataset, and the summarized findings are presented in Table 5. The result of this test brings to light the strengths of the DFViT model, especially maintaining high precision and accuracy across image resolutions. The comparison with other models is very detailed and reveals the relative competitiveness of DFViT in raising the bar higher for malignant cases by its good precision and recall. The detailed analysis underlines the model's robustness and effectiveness in handling the complexities stemming from the classification of histopathological images, hence providing insightful lessons about its practical use in a clinical setting. Also, with different magnification factors of the images, various scales of detail may seriously affect model performance in terms of feature detection and classification from histopathological images. The BreakHis dataset includes four magnification levels: 40×, 100×, 200×, and 400×. Each of them has different resolutions of images that introduce different challenges. Usually, a higher magnification factor introduces noise and complexity while it might carry more useful information for improving the performance in classification. Lower magnifications are less detailed and hence might ease the task, but at the same time, they might miss important features. That our model's performance is consistent across these different magnifications speaks to its strength and ability to generalize; hence, it is well‐suited for clinical applicability, since image resolutions will vary. Further, this variation makes necessary the training of models to cope with different image qualities in order to obtain reliable performances in practical diagnostic tasks.

**TABLE 5:** Average result comparison against BreakHis.

Model	Accuracy	Precision	Recall	F1‐score
Dect [34]	94.12	96.75	89.61	93.04
Deep neural network and XGBoost [37]	91.9	91.5	96.90	94.10
Traditional ML with optimized deep features [38]	95.45	95.45	95.45	95.45
SELF: Stacked ensemble learning [39]	95.1	94	95	94.5
CNN‐SVM with histogram K‐means segmentation [40]	92.6	86.5	93.1	89.79
Proposed model (DFViT)	95.29	97.0	96.91	97.68

##### 40×

**FIGURE 12:**
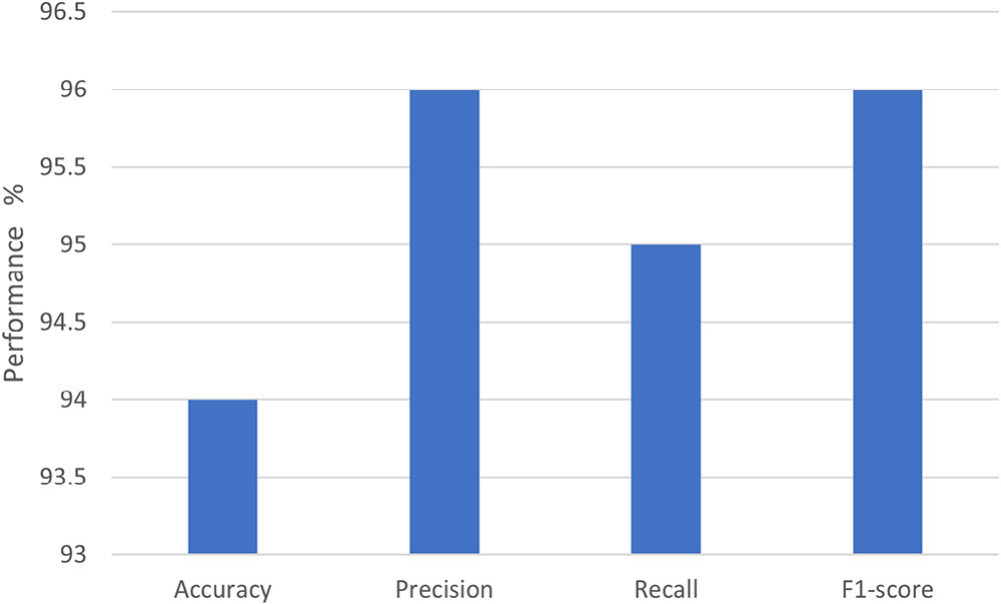
40× results.

The results on 40× images are shown in Figure 12. The bar chart graph shows recall, precision, accuracy, and F1‐score values.

**FIGURE 13:**
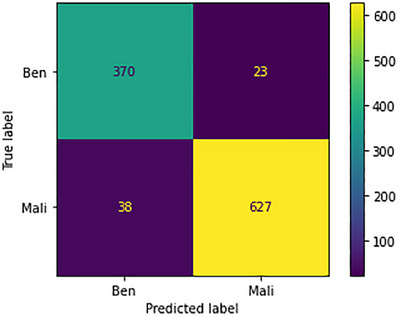
Confusion metrics for 40× images.

The confusion metrics for the 40× images are depicted in Figure 13. Notably, the model's performance surpasses expectations when dealing with the malignant class, as opposed to the benign class. This variance in performance can be attributed primarily to the dataset distribution. Specifically, there's an inherent scarcity of benign images in comparison to their malignant counterparts. Within the set of 120 images belonging to the benign class, a noteworthy observation emerges. Among these, the model falters in prediction for twenty instances. Conversely, the model showcases remarkable proficiency when handling the malignant class. Out of the 665 images representing malignancy, an impressive 627 instances are accurately predicted, attesting to the model's acumen. On a contrasting note, the model makes incorrect predictions for only 38 of these images, further solidifying its competence in this regard.

##### 100×

**FIGURE 14:**
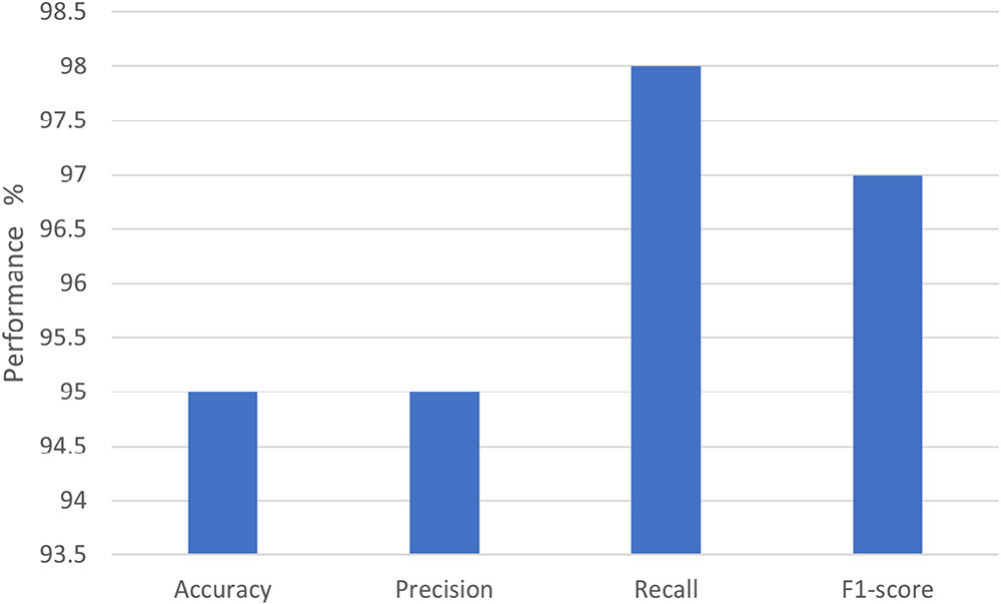
Results for 100× images.

The results on 100× images are shown in Figure 14. The bar chart graph shows accuracy, precision, recall, and F1‐score values.

**FIGURE 15:**
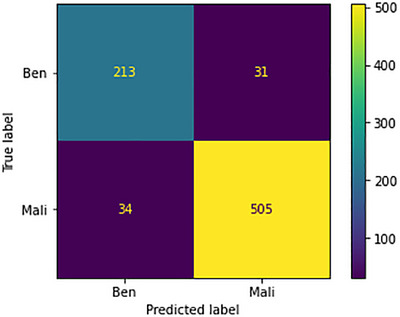
Confusion metrics for 100× images.

The confusion metrics for the 100× images are displayed in Figure 15. The model's performance against the malignant class surpasses its performance against the benign class. The primary reason for this lies in the dataset's distribution, where benign images are significantly outnumbered by malignant images.

Specifically, out of the 244 images belonging to the benign class, there are 31 instances where predictions are inaccurate. In contrast, the model effectively predicts 505 out of 539 malignant class images correctly, with only 34 instances of misprediction. This noteworthy proficiency against the malignant class leads to the model achieving a remarkably high level of accuracy.

##### 200×

**FIGURE 16:**
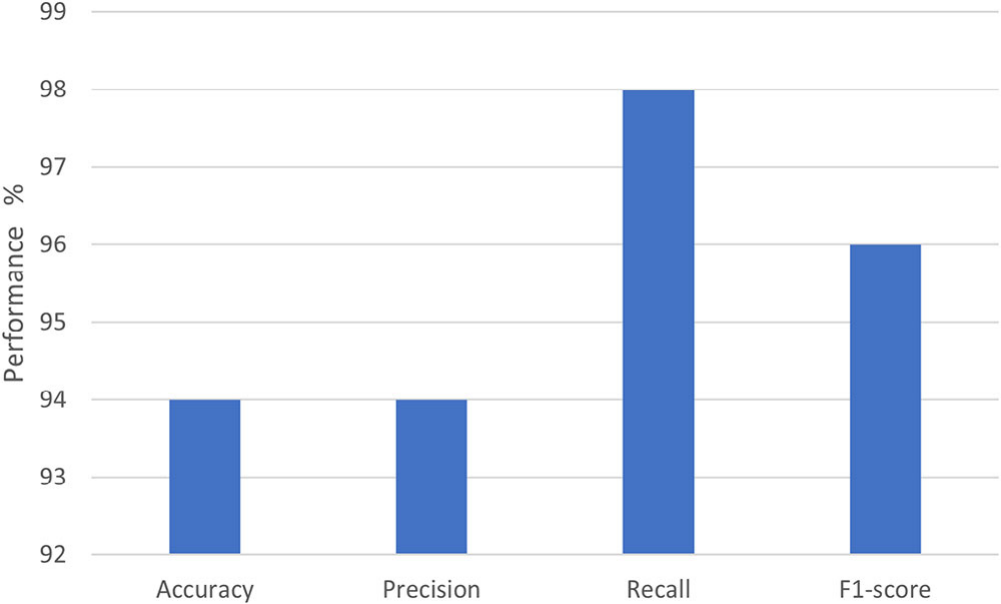
Results for 200× images.

The outcomes derived from the analysis of 200× images are vividly depicted in Figure 16. This illustrative visualization takes the form of a bar chart graph, which effectively encapsulates essential performance metrics such as accuracy, precision, recall, and F1‐score values. By presenting these metrics in a graphical format, the bar chart graph provides a clear and concise overview of the model's performance across different aspects. This visual representation serves as a powerful tool for comprehending how well the model operates and excels in various key evaluation criteria.

**FIGURE 17:**
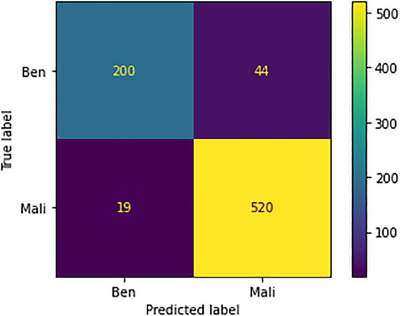
Confusion metrics for 200× images.

The confusion metrics related to the 200× images are visually presented in Figure 17. Within this context, the model's predictive accuracy varies. Specifically, out of the 244 images classified as benign, 44 instances were incorrectly predicted. In contrast, the model demonstrates a significantly high level of proficiency in identifying malignant instances, accurately predicting 520 out of 539 images, with only seven instances bearing incorrect predictions. This exceptional performance underscores the model's efficacy, particularly in distinguishing the malignant class, leading to noteworthy results.

##### 400×

**FIGURE 18:**
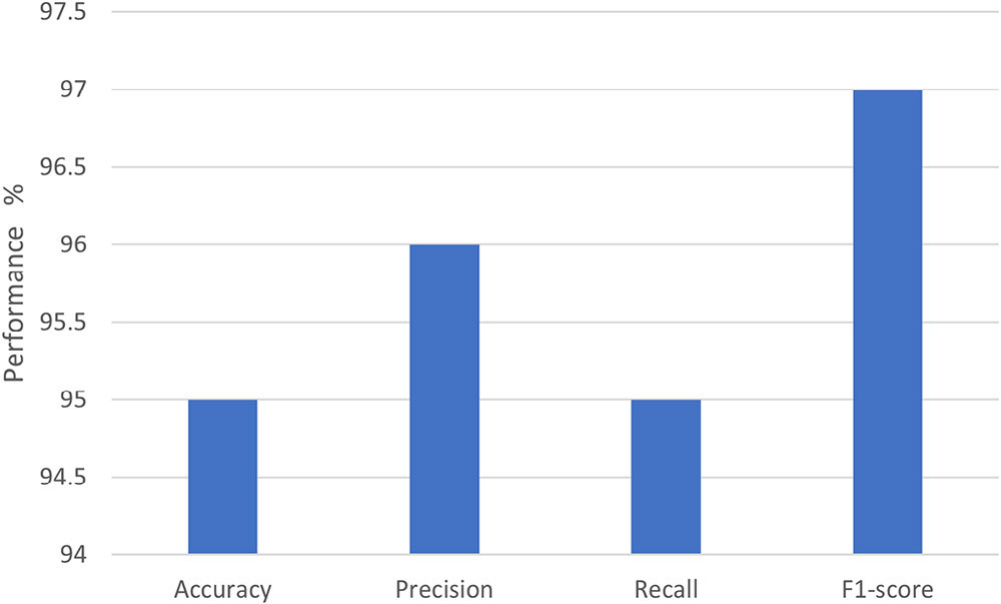
Results for 400× images.

Figure 18 presents the outcomes derived from the analysis of 400× images. This visualization takes the form of a bar chart graph, effectively encapsulating crucial performance metrics such as accuracy, precision, recall, and F1‐score values. Through this graphical representation, the bar chart graph offers a clear and concise overview of how well the model performs across these fundamental evaluation criteria. This visual presentation serves as a valuable tool for comprehending the model's efficacy and proficiency in various key aspects, aiding in the interpretation of its performance on 400× images.

**FIGURE 19:**
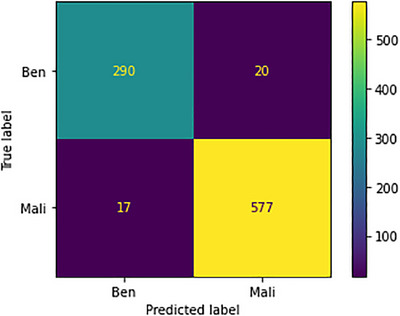
Confusion metrics for 400× image.

The confusion metrics pertaining to the 400× images are visually depicted in Figure 19. Within this context, the model's predictive accuracy exhibits variability. Specifically, out of the 310 images designated as benign, 20 instances were incorrectly predicted. Conversely, the model displays a notably high level of precision when identifying malignant instances, accurately predicting 577 out of 594 images, with only 28 instances bearing incorrect predictions. This remarkable achievement underscores the model's competence, particularly in distinguishing the malignant class, leading to a commendable level of accuracy.

## 6 LIMITATIONS

Even with these novelties, the proposed model has some limitations. The model's tendency to overfit due to its reliance on small datasets is something which needs attention. While innovative, the fusion of RGB and stained images could be prone to noise or complicate the feature extraction process. Also, the increased computational complexity of the Transformer architectures integrated into the CNNs could limit the model's applicability in terms of scalability and efficiency.

## 7 CONCLUSION

This study presented a new deep fusion‐based Vision Transformer model (DFViT), which leveraged higher‐order deep learning to improve classification in histopathological images of breast cancer. The greatest improvements were achieved by the proposed DFViT model, a Transformer architecture with a CNN base, which uniquely combined RGB images with stained ones. This effectively utilized the strengths of both image types, resulting in superior classification performance compared to state‐of‐the‐art Vision Transformers and traditional CNN models. The model's improved performance demonstrated its potential to enhance the accuracy and reliability of breast cancer diagnosis through histopathological image analysis. These findings contribute to the literature by showing how the fusion of different image modalities can significantly boost classification performance. From a practical perspective, the superior results obtained with the DFViT model highlight its potential to enhance breast cancer diagnosis by improving the accuracy and consistency of histopathological image analysis. Key advantages of the DFViT model include better diagnostic precision and reliability, which are critical for successful breast cancer diagnosis. Its ability to utilize both RGB and stained images compensates for the limitations of single‐modality imaging, providing a broader analysis that could have direct clinical applications for early and accurate diagnosis.

## 8 FUTURE RESEARCH

Future research will focus on the enhancement of the classification technique by training baseline models on the Breast Cancer dataset, exploring tailored data augmentation methods to improve the results in consideration of the small size of the dataset, and investigating ensembles of different classifiers and pre‐trained models to enhance the performance of classification of breast cancer histopathology images. In addition, the researchers may work on advanced machine learning and cybersecurity techniques in Internet of Medical Things [48, 49, 50, 51, 52].

## AUTHOR CONTRIBUTIONS

**Ahsan Fiaz**: Conceptualization; methodology; software; writing—original draft; writing—review and editing. **Basit Raza**: Conceptualization; data curation; formal analysis; investigation; project administration; writing—review and editing. **Muhammad Faheem**: data curation; formal analysis; investigation; methodology; writing—review and editing. **Aadil Raza**: Conceptualization; data curation; formal analysis; project administration; resources; supervision; visualization; writing—review and editing.

## CONFLICT OF INTEREST STATEMENT

The authors declare no conflicts of interest.

## Data Availability

The data will be available upon request to the corresponding author.
